# Analysis of epithelial-mesenchymal transition markers in the histogenesis of hepatic progenitor cell in HBV-related liver diseases

**DOI:** 10.1186/s13000-016-0587-y

**Published:** 2016-11-24

**Authors:** Wei Xu, Nong-Rong Wang, Hua-Feng Wang, Qiong Feng, Jun Deng, Zhi-Qiang Gong, Jian Sun, Xiao-Liang Lou, Xue-Feng Yu, Lv Zhou, Jin-Ping Hu, Xiao-Feng Huang, Xiao-Qing Qi, Yan-Juan Deng, Rui Gong, Yan Guo, Meng-Meng Wang, Jia-Cheng Xiao, Huan Deng

**Affiliations:** 1 Department of General Surgery, Jiangxi Children’s Hospital, Nanchang, China; 2 Molecular Medicine and Genetics Center, The Fourth Affiliated Hospital of Nanchang University, Nanchang, China; 3 Department of Gastroenterology, The Fourth Affiliated Hospital of Nanchang University, Nanchang, China; 4 Department of Pathology, Ruijin Hospital, School of Medicine, Shanghai Jiaotong University, Shanghai, China; 5 Department of Pathology, The Second Affiliated Hospital of Nanchang University, Nanchang, China; 6 Renmin Institute of Forensic Medicine in Jiangxi, Nanchang, China; 7 Emergency Department, The First Affiliated Hospital of Nanchang University, Nanchang, China; 8 Department of Pathology, The Fourth Affiliated Hospital of Nanchang University, Nanchang, China; 9 Department of Pathology, Tenth People’s Hospital of Tongji University, Shanghai, China; 10 Department of Pathology, The Fourth Affiliated Hospital of Nanchang University, 133 South Guangchang Road, Nanchang, 330006 Jiangxi China

**Keywords:** Hepatic progenitor cell, Epithelial-mesenchymal transition, HBV, Cholangiocyte, Histogenesis

## Abstract

**Background:**

The origin and heterogeneity of hepatic progenitor cells (HPCs) remain unclear. This study aimed to investigate the involvement of epithelial-mesenchymal transition (EMT) in the histogenesis of HPCs.

**Methods:**

Surgical liver specimens from patients with HBV-related hepatitis and cirrhosis were investigated with double immunofluorescence labeling to detect antigens associated with HPCs and EMT. Ductular reactions were subjected to quantitative reverse transcription PCR following isolation by laser capture microdissection. Electron microscopic examination was performed to find an ultrastructural evidence of EMT.

**Results:**

The number of EpCAM-positive HPCs was proportional to the disease severity. The S100A4 expression of HPCs was firstly observed in mild hepatitis and increased significantly in moderate hepatitis, but decreased in severe hepatitis and cirrhosis. The levels of MMP-2, Twist, and Snail increased in direct proportion to the number of HPCs. Some hepatocytes adjacent to portal tracts in cirrhosis showed positivity for MMP-2. Although CK7 and E-cadherin levels decreased in mild and moderate hepatitis, HPCs re-expressed both of them in severe hepatitis and cirrhosis. However, HPCs expressed neither vimentin nor αSMA. The relative mRNA expression levels of EpCAM and EMT-associated markers supported immunohistochemical results. Electron microscopic examination demonstrated the existence of intercellular junctions among HPCs, cholangiocytes, and intermediate hepatocyte-like cells.

**Conclusion:**

We provided preliminary evidence for the involvement of EMT in the histogenesis of HPCs from cholangiocytes in HBV-related liver diseases. HPCs may re-transdifferentiate into hepatocytes, and the differentiation direction depends, at least in part, on interactions between HPCs and the surrounding microenvironment, especially the non-resolving inflammation caused by HBV infection.

## Background

It is well known that terminal stage liver diseases can be treated by liver transplantation with relatively good five-year survival. However, limited donor organs and rejection reactions restrict its usage. Thus, it is necessary to establish a novel strategy. Hepatic progenitor cells (HPCs)-based therapy is considered to be a promising replacement for liver transplantation because HPCs are capable of differentiating into both hepatocyte and biliary lineage [1, 2]. However, this therapeutic strategy cannot be fully exploited until the mechanisms underlying the histogenesis of HPCs are clearly elucidated. It is traditionally assumed that HPCs originate in the smallest ramifications of the bile ducts or in locations outside the liver, such as bone marrow (BM) [3–5]. However, liver stem cells have never been observed in the adult liver. Meanwhile, the restricted potential to differentiate into hepatocytes and cholangiocytes also qualifies HPCs as true stem cells [6]. Recent studies suggested that EMT is involved in the pathogenesis of liver cirrhosis, a common endpoint of most chronic liver diseases. There is now convincing evidence that TGF-β1 can induce both cultured hepatocytes and cholangiocytes to undergo EMT [7–9]. Notably, some cells undergoing EMT/MET (mesenchymal-epithelial transition) can simultaneously express epithelial/mesenchymal markers and HPCs markers [10]. Furthermore, they can repair injured liver in rodent models [11], raising the exciting possibility that HPCs may represent a subpopulation of cells undergoing EMT/MET [12]. However, most available evidence supporting this hypothesis is indirect and obtains from rodent models or in vitro studies.

The EMT is a dynamic process that cannot be followed serially in humans because of technical limitations. In this study, we examined specimens from patients with benign HBV-related diseases, including hepatitis and cirrhosis. We used double immunofluorescence staining and RT-qPCR (quantitative reverse transcription PCR) to detect characteristic markers that are associated with HPCs, EMT, and epithelial/mesenchymal cells. In this study, EpCAM was used for identification of HPCs, because several studies indicated that EpCAM is capable of relative higher specificity than other HPCs markers such as CD133, CK19, and OV-6 [13–15]. S100A4, the human homolog of murine fibroblast-specific protein 1 (FSP1), has been demonstrated to be a key marker of early fibroblast lineage development and EMT [16, 17]. Consequently, loss of cytokeratin (CK) and adherens junction components such as E-cadherin promotes the detachment of transitioning cells from primary sites [18, 19]. At later stages, EMT is accompanied by an increase in motility and matrix invasion, which is consistent with elevated levels of vimentin and matrix metalloproteinases (MMPs) [19]. EMT-derived fibroblast cells can express α-smooth muscle actin (αSMA) [20]. Meanwhile, several nuclear transcription factors contribute to the EMT cascade. Twist, a known helix-loop-helix transcription factor, can directly affect its down-stream element Snail to suppress the expression of E-cadherin [21–23].

## Methods

### Patients and clinical data

Sixty patients (42 men and 18 women) with HBV-related diseases were enrolled. Serum HBV DNA should be ≥2.5 pg/mL and last at least 3 months. Fifteen samples obtained from liver donors served as control. A part of each sample was snap-frozen and stored at −80 °C until being used for laser capture microdissection (LCM). Clinical data were recorded (Table 1). All patients gave written informed consent to participate in the study in accordance with the Helsinki Declaration, and this study was approved by the Regional Ethics Committee (Medical Ethics Committee of The Fourth Affiliated Hospital of Nanchang University and Ruijin Hospital, School of Medicine, Shanghai Jiaotong University).

**Table 1:** Clinical, biochemical and histopathological characteristics of patients with HBV associated diseases

Case	Sex	Age (years)	Disease	AST (IU/L)	ALT (IU/L)	Albumin (g/L)	Total bilirubin (μmol/L)	Platelet (×10^9^/L)	Serum AFP (n/g/mL)
1	M	28	Hepatitis (Mild)	25	20	35	15.7	62	5.8
2	M	49	Hepatitis (Mild)	17	22	38	19.3	321	5.2
3	F	27	Hepatitis (Mild)	18	31	30	31.9	158	10
4	F	58	Hepatitis (Mild)	21	11	41	19.2	71	3.39
5	M	56	Hepatitis (Mild)	21	28	41	12.4	240	9.01
6	M	50	Hepatitis (Mild)	33	25	59	28.5	257	5.2
7	M	39	Hepatitis (Mild)	18	15	24	12	158	3.2
8	M	24	Hepatitis (Mild)	21	19	36	10.5	182	6.82
9	M	28	Hepatitis (Mild)	39	50	41	22.9	234	4.45
10	M	32	Hepatitis (Mild)	25	26	39	12.3	120	5.3
11	M	36	Hepatitis (Mild)	14	16	41	19.3	178	4.3
12	M	28	Hepatitis (Mild)	24	18	28	5.6	205	5.25
13	M	22	Hepatitis (Mild)	30	24	39	23.1	124	2.8
14	M	42	Hepatitis (Mild)	27	31	29	22.2	94	9.4
15	F	47	Hepatitis (Mild)	22	23	44	16	322	2.6
16	F	31	Hepatitis (Moderate)	43	41	28	21.3	167	10.2
17	F	38	Hepatitis (Moderate)	30	29	41	22.4	203	4.2
18	M	39	Hepatitis (Moderate)	18	17	30	21.4	205	6.2
19	M	41	Hepatitis (Moderate)	25	23	35	29	231	2.9
20	M	32	Hepatitis (Moderate)	31	29	27	17.4	160	1.84
21	M	28	Hepatitis (Moderate)	40	47	42	13.4	205	9.2
22	M	57	Hepatitis (Moderate)	22	19	42	21.8	265	4.28
23	F	41	Hepatitis (Moderate)	25	30	37	10.8	46	4.38
24	F	57	Hepatitis (Moderate)	21	12	19	31.3	254	3.21
25	M	39	Hepatitis (Moderate)	28	36	32	14.8	269	3.42
26	M	24	Hepatitis (Moderate)	39	21	57	19.9	189	3.02
27	M	60	Hepatitis (Moderate)	21	14	30	12.4	94	3.59
28	F	25	Hepatitis (Moderate)	32	21	51	20	109	2.5
29	F	39	Hepatitis (Moderate)	23	26	41	21.3	206	11.7
30	M	42	Hepatitis (Moderate)	48	35	44	25.1	207	8.2
31	F	39	Hepatitis (Severe)	54	92	26	35	307	10.6
32	M	52	Hepatitis (Severe)	35	37	18	15	184	1.23
33	M	36	Hepatitis (Severe)	23	71	39	23.1	159	9.08
34	M	21	Hepatitis (Severe)	35	49	25	38.1	93	3.57
35	M	31	Hepatitis (Severe)	28	58	62	29.5	222	4.9
36	M	39	Hepatitis (Severe)	23	31	31	16.6	81	2.2
37	M	26	Hepatitis (Severe)	18	24	50	20.7	152	8.1
38	F	27	Hepatitis (Severe)	29	42	41	29.1	121	2.7
39	M	40	Hepatitis (Severe)	7	15	25	15.2	106	3.9
40	M	47	Hepatitis (Severe)	37	27	31	8.2	83	4.6
41	M	33	Hepatitis (Severe)	24	62	28	43.2	119	3.12
42	M	51	Hepatitis (Severe)	16	36	32	24.3	132	7.9
43	M	32	Hepatitis (Severe)	41	61	18	16.8	182	10
44	F	41	Hepatitis (Severe)	66	34	30	38.7	204	4.33
45	M	25	Hepatitis (Severe)	20	15	50	23.4	310	3.8
46	F	51	non-HCC cirrhosis	12	41	48	36.2	49	9.2
47	M	47	non-HCC cirrhosis	31	21	42	36.9	69	4.9
48	M	38	non-HCC cirrhosis	27	33	26	13.9	891	4.27
49	M	40	non-HCC cirrhosis	47	36	40	26.1	104	21.34
50	F	41	non-HCC cirrhosis	30	14	28	40.3	71	3.6
51	M	55	non-HCC cirrhosis	38	10	28	28.4	158	6.87
52	F	60	non-HCC cirrhosis	20	51	32	11.4	175	5.74
53	M	49	non-HCC cirrhosis	44	27	35	19.3	92	3.56
54	M	54	non-HCC cirrhosis	20	42	21	14.3	60	7.24
55	M	59	non-HCC cirrhosis	25	26	46	18.5	127	2.3
56	M	48	non-HCC cirrhosis	19	15	39	21.2	115	6.8
57	F	32	non-HCC cirrhosis	307	342	49	29.3	110	7.6
58	M	56	non-HCC cirrhosis	22	19	26	20.6	172	8.3
59	F	53	non-HCC cirrhosis	31	26	24	18.2	77	2.48
60	F	47	non-HCC cirrhosis	20	25	51	21.9	51	3.9

### Histopathological examination

The histopathological evaluation of all sections was performed by two independent researchers (JH Mei and YQ Xiao) who were blinded regarding patient details. The method proposed by Bedossa et al. was served as grading standard [24] (Table 2).

**Table 2:** Histological activity of liver biopsy of chronic hepatitis B

Histological Activity	Histological Feature
Mild (A1)	PMN = 0 LN = 1
	PMN = 1 LN = 0, 1
Moderate (A2)	PMN = 0 LN = 2
	PMN = 1 LN = 2
	PMN = 2 LN = 0, 1
Severe (A3)	PMN = 2 LN = 2
	PMN = 3 LN = 0, 1, 2

PMN, piecemeal necrosis; 0, none; 1, mild; 2, moderate, 3, severe; LN, lobular necrosis; 0, no or mild; 1, moderate; 2, severe

### Double immunofluorescence labeling and morphometric determinations

The formalin fixed and paraffin embedded tissues were cut at 2- to 5-μm and retrieved in citrate buffer (pH 6.0) at 95 °C −99 °C for 15 min. These slides were then blocked with 20% BSA before the incubation with a cocktail for EpCAM and one of the EMT-associated markers at 4 °C overnight (Table 3). Two different antibodies for EpCAM were employed in this study to avoid cross-reactions (Table 3). Serial sections from three breast ductal carcinoma samples served as the positive control to compare the sensitivity and specificity of the two different EpCAM antibodies according to manufactory’s instruments. Two independent pathologists, who were blinded regarding staining details, evaluated and recorded the extent and intensity of sections respectively by using the following arbitrary scale: 0: ≤ 25%, no staining; 1: 26–50%, weak staining; 2: 51–75%, moderate staining; 3: 76–100%, strong staining. Statistical analysis based on the accumulated scores suggested that there were no significant differences between the two antibodies (*p* < 0.01). FITC or TRITC-conjugated secondary antibodies (Jackson Immunoresearch, USA) were incubated with the pretreated slides at room temperature for 60 min. The dilutions were 1:100 and 1:150, respectively. Finally, the slides were washed with PBS for three times and mounted with anti-fade medium (Vector, USA). A Nikon 80i microscope with the Intensilight fluorescence set and DS-Ri2 camera (Nikon, Japan) was used to perform the image analysis.

**Table 3:** Summary of primary antibody used for immunohistochemistry

Antibody	Isotype	Supplier	Dilution
EpCAM	IgG1(rabbit)	Epitomics	1:300
EpCAM	IgG1(mouse)	abcam	1:100
Cytokeratin 7	IgG1(mouse)	DAKO	1:100
E-Cadherin	IgG1(mouse)	DAKO	1:50
S100A4	IgG(rabbit)	abcam	1:50
MMP-2	IgG(rabbit)	abcam	1:250
Twist	IgG1(mouse)	abcam	1:150
Snail	IgG(rabbit)	abcam	1:100
Vimentin	IgG1(mouse)	DAKO	1:100
α-SMA	IgG2a(mouse)	DAKO	1:100

HPCs were considered to be small to medium EpCAM-positive cells localizing within ductular reactions (DRs). Counting of positive cells was conducted on sections with an immunofluorescence microscope. The corresponding number of non-overlapping fields in the section was counted using the 40× objective. The average number of positive cells in each square centimeter was calculated for each specimen.

### LCM and RT-qPCR

Frozen sections were placed onto PET-membrane slides (Leica Microdissect, Herborn, Germany) and briefly stained with hematoxylin and eosin (H&E). DRs were dissected using LCM with a Leica SVS LMD System (Leica Microsystems, Wetzlar, Germany). A sufficient number of cells were obtained from 5 to 10 tissue sections. RNA was isolated using an RNeasy Micro Kit (Qiagen, Hilden, Germany) and then evaluated by the Agilent 2100 bioanalyzer (Agilent Technologies, Palo Alto, CA, USA) as in a previous study [13]. RT-qPCR was performed with a Select cDNA Synthesis Kit (Bio-Rad, California, USA). Relative mRNA levels of targeted genes were measured by RT-qPCR during 40 cycles and expressed as ΔCt values when compared to GAPDH (primer sequences see Table 4) [25].

**Table 4:** The sequences of primers used in qRT-PCR

Gene	Sense	Antisense	Product (bp)
EpCAM	CTGGACTGGAATGCTGAG	CGAAGATGACGATGAGGAT	178
S100A4	GATGAGCAACTTGGACAGCAA	CTGGGCTGCTTATCTGGGAAG	123
MMP-2	CCGTCGCCCATCATCAAGTT	CTGTCTGGGGCAGTCCAAAG	169
Twist	GAATGACCGCTTCGCCAACTA	CCGCATCTCCTCCTCGTAGA	136
Snail	ACTACTGCTGAGCGTGAGATTG	CGATGAAGGATGGCTGGAACA	199
CK7	TTGTGGTGCTGAAGAAGG	CTGTCAACTCCGTCTCATT	123
E-cadherin	CGAGAGCTACACGTTCACGG	GTGTCGAGGGAAAAATAGGCTG	117
Vimentin	GACGCCATCAACACCGAGTT	CTTTGTCGTTGGTTAGCTGGT	238
αSMA	GTGTTGCCCCTGAAGAGCAT	GCTGGGACATTGAAAGTCTCA	109
GAPDH	GAAGATGGTGATGGGATTTC	GAAGGTGAAGGTCGGAGT	230

### Transmission electron microscopic (EM) examination

All tissues were fixed in 4% glutaraldehyde in 0.1 m phosphate buffer pH 7.3 and embedded in Epon 812. Following double staining with uranyl acetate and lead citrate, the sections were examined using a Philips CM120 transmission EM.

### Statistical analysis

Continuous normally distributed variables are represented as mean ± SD. The inflammatory grade was summarized by the median. ANOVA was used to evaluate the difference among the comparative mRNA expression of each gene in normal, hepatitis and cirrhosis specimens. The LSD-*t* test was applied for post-hoc comparisons. *P <* 0.05 was considered statistically significant. Data analyses were carried out using SPSS version 13.0 for Windows (SPSS, Inc., Chicago, USA).

## Results

### Expression of EMT markers in HBV-related liver diseases

**Fig. 1:**
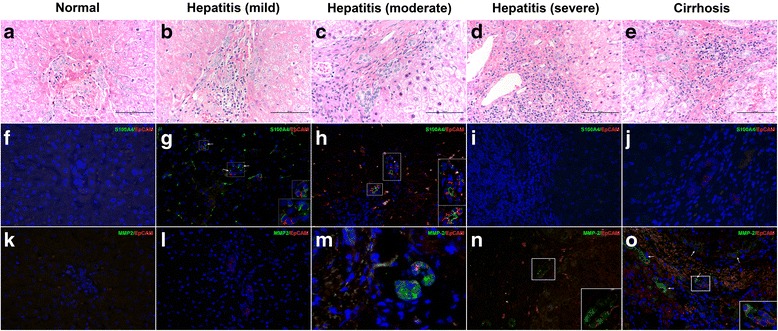
The expressions of S100A4 and MMP-2 in HPCs. **a**, **f**, **k** In sections from normal liver, parenchymal cells expressed neither S100A4 nor MMP-2 (original magnification × 200). **b**, **g**, **l** Some HPCs in mild hepatitis showed immunopositivity for EpCAM, rather than MMP-2. (original magnification × 200, inserts, original magnification × 400). **c**, **h**, **m** Majority of HPCs in moderate hepatitis expressed strong-diffuse cytoplasmic positivity for S100A4 and MMP-2 (original magnification × 200, inserts, original magnification × 400). **d**, **i**, **n** The expression level of S100A4 decreased significantly in HPCs from severe hepatitis. But HPCs expressed a high level of MMP-2 (original magnification × 200, inserts, original magnification × 400). **e**, **j**, **o** HPCs in cirrhosis sections showed negativity for S100A4. Both HPCs and intermediate hepatocytes (*arrow*) adjacent to portal tracts expressed MMP-2 (original magnification × 200)

**Fig. 2:**
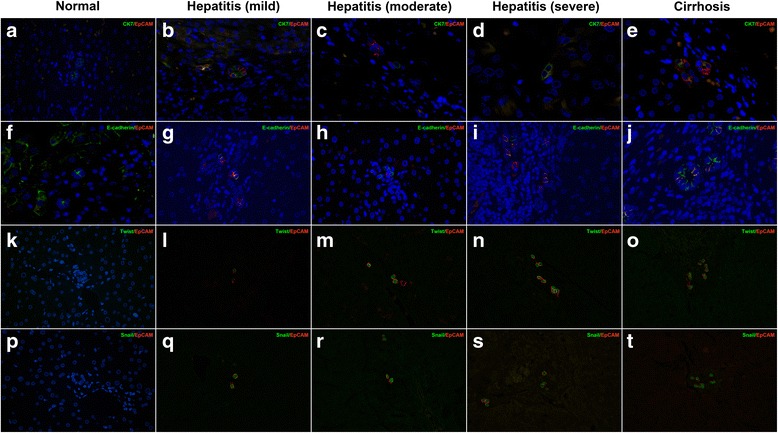
The expressions of CK7 and E-cadherin in HPCs. **a**, **f** Cholangiocytes in normal liver exhibited positivity for CK7 and E-cadherin rather than EpCAM (original magnification × 200). **b**, **g** HPCs within DRs of mild hepatitis co-expressed CK7 and EpCAM. The down-regulated expression of E-cadherin was detected in DRs of cirrhosis (original magnification × 200). **c**, **h** The number of CK7- or E-cadherin-positivity HPCs decreased significantly in moderate hepatitis (original magnification × 200). **d**, **i** In sections of severe hepatitis, the number of CK7- or E-cadherin-positivity HPCs increased (original magnification × 200). **e**, **j** Majority of HPCs within DRs of cirrhosis re-expressed CK7 or E-cadherin (original magnification × 200). **k**, **p** Cholangiocytes and hepatocytes in the normal liver did not express Twist and Snail (original magnification × 200). **l**-**o** Twist-positive HPCs numbers increased in direct proportion to disease severity (original magnification × 200). **q**-**t** Hepatitis and cirrhosis resulted in the elevated level of Snail (original magnification × 200)

In normal liver, DRs were absent and cholangiocytes in intrahepatic bile duct showed positivity for CK7 and E-cadherin (Figs. 1a, and 2a, f). They did not express EpCAM or EMT-associated markers (Figs. 1f, k and 2k, 2p).

Although a definite phenotypic marker has not yet been developed, S100A4 has been described as one of the earliest makers and activators of EMT [17]. In sections from patients with mild and moderate inflammation, some but not all S100A4-positive cells expressed the HPC marker EpCAM (*n* = 1.19 ± 0.68 per cm^2^ and *n* = 3.09 ± 0.52 per cm^2^, respectively) (Fig. 1g, h). Noteworthy, only a few of HPCs in sections of severe inflammation and cirrhosis expressed S100A4 (*n* = 1.02 ± 0.44 per cm^2^ and *n* = 0.26 ± 0.17 per cm^2^) (Fig. 1i, j).

MMPs comprise a large family that regulates essential steps of embryogenesis and wound healing. Our results showed that HPCs and hepatocytes in sections of mild hepatitis were negative for MMP-2 (Fig. 1l). In moderate and severe hepatitis, a portion of HPCs expressed MMP-2 (*n* = 2.45 ± 0.98 per cm^2^ and *n* = 2.45 ± 0.98 per cm^2^, respectively) (Fig. 1m, n). Some intermediate hepatocyte-like cells (IHLCs) adjacent to portal tracts from patients with cirrhosis showed immunopositivity for MMP-2 rather than EpCAM (Fig. 1o).

The proteomic features of EMT also include the loss of epithelial markers. Although almost all of HPCs within DRs of mild hepatitis co-expressed CK7 and E-cadherin (*n* = 1.77 ± 0.83 per cm^2^ and *n* = 1.42 ± 0.76 per cm^2^, respectively), a few of HPCs displayed neither of them (Fig. 2b, g). The number of CK7- or E-cadherin-positive HPCs decreased significantly in moderate hepatitis as compared to mild hepatitis (*n* = 0.65 ± 0.33 per cm^2^ and *n* = 0.78 ± 0.45 per cm^2^, *P* < 0.05) (Fig. 2c, h). Noteworthy, HPCs expressed elevated levels of CK7 and E-cadherin in severe hepatitis and cirrhosis as compared to mild hepatitis (*n* = 2.11 ± 0.64 per cm^2^, 3.08 ± 0.52 per cm^2^, and *n* = 1.87 ± 0.56 per cm^2^, 3.18 ± 0.49 per cm^2^, *P* < 0.05) (Fig. 2d, e, i, and j).

Repression of E-cadherin by transcriptional regulators such as Twist and Snail serves as a key event during EMT [26]. Nuclear positivity for Twist or Snail in HPCs was firstly detected in sections of mild hepatitis (*n* = 1.62 ± 0.49 per cm^2^ and *n* = 1.70 ± 0.52 per cm^2^, respectively) (Fig. 2l, q), and the number of Twist- or Snail-positive HPCs was proportional to the severity of HBV-related disease (*n* = 2.65 ± 0.83, 3.79 ± 0.33, 4.91 ± 0.39 per cm^2^ and *n* = 2.41 ± 0.45, 3.66 ± 0.81, 4.78 ± 0.53 per cm^2^, respectively, *P* < 0.05).

The expression of αSMA and vimentin during EMT indicates the transdifferentiation from epithelial cells into mature mesenchymal cells such as myofibroblasts. In sections of normal, hepatitis, and cirrhotic liver, HPCs, and parenchymal cells did not express αSMA or vimentin (data not show).

### Comparison of mRNA expression of EMT-associated markers

**Fig. 3:**
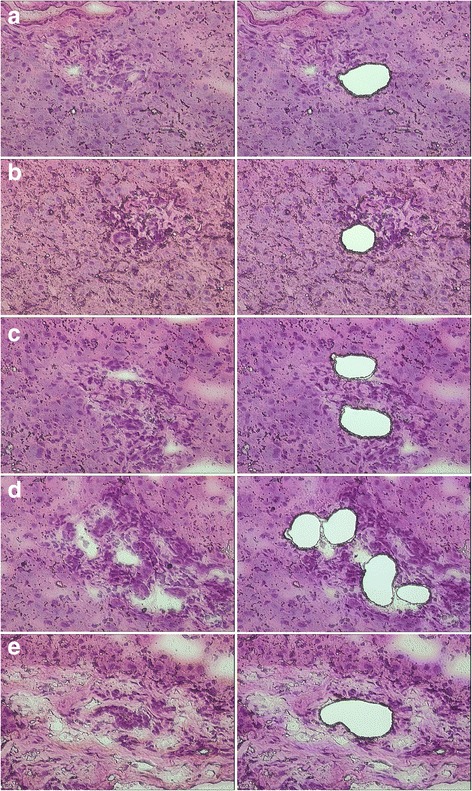
Hematoxylin and eosin staining of liver tissue sections before (*left*) and after (*right*) laser capture microdissection. **a** Bile ducts or DRs were isolated from tissue sections of normal liver, **b** mild hepatitis, **c** moderate hepatitis, **d** severe hepatitis, and **e** cirrhosis

**Fig. 4:**
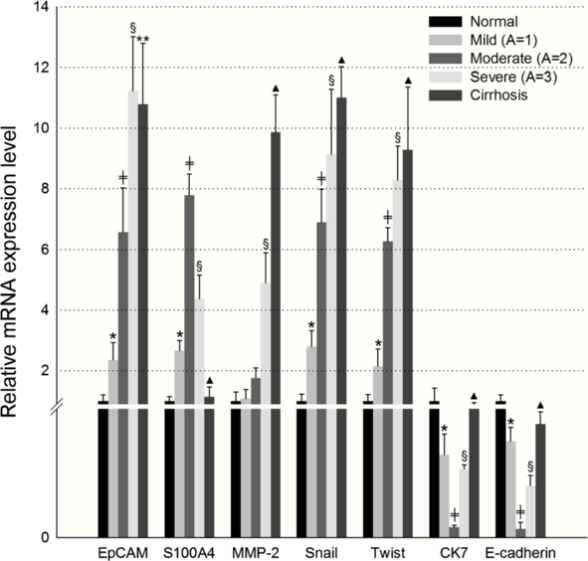
mRNA expression levels of HPCs after tissue picking by laser capture microdissection. The expression levels of EpCAM, S100A4, MMP-2, CK7, E-cadherin, Twist, and Snail were detected by RT-qPCR. * mild hepatitis versus normal control, *P* <0.05; ╪ moderate hepatitis versus mild hepatitis, *p* <0.05; § severe hepatitis versus moderate hepatitis, *P* < 0.05; ▲ cirrhosis versus severe hepatitis, *P* < 0.05; ** cirrhosis versus severe hepatitis, *P* > 0.05

We employed LCM to collect DRs (Fig. 3). Total RNA extracted from the microdissected samples was 37–102 ng. Consistent with the double immunofluorescence staining results, RT-qPCR showed that normal bile ducts did not show aberrant expression of EpCAM nor EMT-associated markers. The elevated mRNA levels of EpCAM, S100A4, Twist, or Snail were firstly confirmed in mild hepatitis and showed a positive relationship with inflammation severity. S100A4 mRNA expression in severe inflammation liver specimens was significantly higher than that in cirrhotic liver (*P* < 0.001) (Fig. 4). The expression of MMP-2 increased significantly in moderate, severe hepatitis and cirrhotic liver (*P* < 0.001). DRs in HBV-related diseases did not express vimentin and αSMA. The reduction in mRNA expression of the epithelial markers CK7 and E-cadherin was detected in mild and moderate hepatitis (*P* < 0.001). However, their expression levels increased and paralleled the severity of diseases in severe hepatitis and cirrhosis (*P* < 0.001) (Fig. 4).

### Ultrastructural findings

**Fig. 5:**
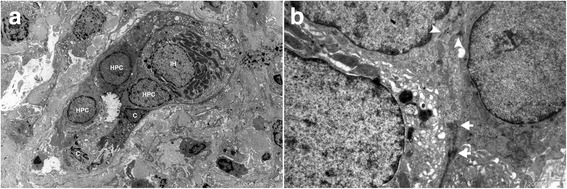
EM examination was performed to find ultrastructural evidence for the transition. **a** Three HPCs identified by EM. An IHLC with more cytoplasm and mitochondria than HPCs was in close association with HPCs. **b** There were intercellular junctions among HPCs, cholangiocytes, and intermediate hepatocyte (*arrow*). Tonofilament bundles in cytoplasm of HPCs indicated the reacquisition of epithelial characteristic (*arrowhead*)

HPCs, characterized by their oval shape, small size (7 to 15 μm in diameter) and scanty electron-dense cytoplasm, existed in all specimens of HBV-related diseases (Fig. 5a). Intercellular junctions were noted between HPCs and some IHLCs. The latter is characterized by abundant cytoplasm and mitochondria, fewer tonofilament bundles compared with HPCs (Fig. 5a, b). The cellular junctions were also observed between HPCs, cholangiocytes, and intermediate hepatocyte-like cells, suggesting a transdifferentiation from mature cholangiocyte to immature hepatocyte (Fig. 5a). Additionally, HPCs at the portal tract/hepatocyte interface contained a greater number of tonofilament bundles than HPCs in the periportal tract, suggesting the intriguing possibility of epithelial phenotype reacquisition (Fig. 5b).

## Discussion

Despite recent advances in pathophysiology, the histogenesis and heterogeneity of HPCs in humans are matters of debate. In this study, we combined data from immunophenotypic and mRNA studies with ultrastructural examination to provide preliminary evidence for the involvement of EMT in the histogenesis of HPCs in HBV-related liver diseases.

S100A4, which modifies cell motility and growth through interactions with the cytoskeleton and the C-terminus of p53, has been proposed as an early marker of EMT [17]. Co-expression of EpCAM and S100A4 provided clear evidence that although these HPCs have an epithelial phenotype, they are actively engaged in EMT. HPCs also expressed another important EMT marker MMP-2, which possesses the ability to degrade basement membrane and increases cell motility [27]. The activity of MMPs can alter the expression of E-cadherin and vimentin and promote the EMT process [28]. High levels of MMP-2 may promote the dispatch of HPCs from DRs.

A hallmark of EMT is the aberrant expression of E-cadherin (encoded by *CDH1*), which is always linked to the tumorigenesis of many epithelial cancers [29]. E-cadherin is a key factor of cell-cell adhesion junctions in the maintenance of cell polarity and structure. Recent studies uncovered its critical roles in proliferation, differentiation, and carcinogenesis. Analogous to cholangitis, the loss of E-cadherin in the liver contributes to the periportal inflammation and later periductal fibrosis [30]. A study making use of a transgenic mouse model where the expression of cre was directed by the *albumin* enhancer/promoter (termed Alb-cre) demonstrated that these lesions arose as a consequence of the loss of E-cadherin in the cholangiocytes [30]. The reexpression of cre in hepatocytes could not attenuate the effects [31, 32]. In this study, the expression of E-cadherin and CK7 in HPCs decreased significantly from mild hepatitis to moderate hepatitis, revealing that these transitioning cells might derive from epithelial cells within DRs and were losing cell-cell contacts. However, the number and ratio of E-cadherin- or CK7-positive HPCs increased in sections of severe hepatitis and cirrhosis, suggesting that the transitioning cells might reverse the cascade and reacquire epithelial characteristics.

Twist is a core element during EMT process [22]. The overexpression or promoter methylation of Twist is always associated, in a statistically significant manner, with the tumor aggressiveness [33–35]. Activated Twist binds to the promoter region of E-cadherin and transcriptionally downregulates E-cadherin expression [36]. Together with the polycomb protein Bmi1, Twist contributes to the stemness of cells, which is one of the most important features of HPCs [22].

Snail is a zinc-finger transcription factor, which can induce EMT by repression of E-cadherin [23]. TGF-β promotes EMT by up-regulating Snail expression via a Smad-dependent pathway. Snail forms a transcriptional repressor complex with SMAD3/4. The complex then targets the adjacent E-boxes and Smad-binding elements in genes encoding junction proteins such as E-cadherin, CAR and occluding [37].

In all hepatitis and cirrhotic sections, the majority of HPCs within DRs or bile ducts expressed high levels of Twist or Snail, which are proportional to the severity of HBV-related diseases. RT-qPCR further validated these findings, indicating that Twist and Snail were involved in the EMT process of HPCs via the repression of E-cadherin in HBV-related diseases.

We tried to obtain further evidence for the transdifferentiation from HPCs into mature mesenchymal cells by using antibodies for αSMA and vimentin. However, neither cholangiocytes nor hepatocytes expressed αSMA or vimentin in this study. We considered two possibilities for this situation. First, αSMA- or vimentin-positive HPCs may detach from DRs because of an increase in motility and matrix invasion [38]. Hence, these transitioning cells at an advanced stage of EMT do not express EpCAM and detach from DRs. Second, as reversibility is an important feature of EMT, it is tempting to speculate that these transitioning cells may re-transdifferentiate into parenchymal cells through MET under certain conditions.

Although we did not obtain substantial results about whether hepatocytes could transdifferentiate into HPCs through EMT, some periportal intermediate hepatocytes in sections from cirrhotic livers showed immunopositivity for MMP-2. We postulated an intriguing possibility that, at least in HBV-related liver diseases, intermediate hepatocytes may represent daughter cells of HPCs rather than a cellular origin of HPCs. Ultrastructural evidence that intercellular junctions existed between HPCs and intermediate hepatocytes further supported this hypothesis. Consistent with our findings, a recent study based on an established rodent model provided convincing evidence for challenging the concept that hepatocytes can acquire a mesenchymal phenotype in vivo via EMT [39]. In contrast, previous studies demonstrated that hepatocytes may also be capable of undergoing EMT in vitro [40, 41]. Furthermore, an elaborate study indicated that the Hippo signal pathway can direct hepatocytes to transdifferentiate into HPC-like cells and finally mimic an atypical ductular reaction [42]. We believe that different microenvironments may result in different outcomes. Most recently, an elaborate study provided supporting evidence [43]. Foetal HPCs can form hepatic cysts characterized by Albumin-positive/CK19-negative in vitro. However, if foetal HPCs are pre-cultured on gelatin-coated dishes, they are capable of forming cholangiocytic cysts expressing Albumin-negative/CK19-positive, similar to that of HPCs [43]. This cholangiocytic cysts formation can be hampered by hepatic maturation factors, such as hepatocyte growth factor (HGF) and oncostatin M (OSM). Of note, the study also indicated that TGF-β antagonist A-8301 plays an important role in the cholangiocytic cysts formation. It seems that this conclusion is consistent with our hypothesis and observations in vivo. TGF-β derived from portal parenchymal cells or infiltrating immune cells promotes the entrance of cholangiocytes into the EMT cascade and transdifferentiation into hepatocyte-like cells. However, further experiments will be required to gain more insights into the regulatory mechanisms.

Chronic HBV infection may serve as a powerful driver of EMT. The regeneration capacity of mature hepatocytes is overwhelmed during massive or chronic liver infection. Various cytokines released from injured parenchymal cells establish a stable inflammatory infiltrate through the recruitment of leukocytes from blood [44]. T-cells mediate both liver injury and viral clearance in animal models of HBV infection and produce regulatory cytokines such as TGF-β, a powerful driver of EMT [45–47]. Furthermore, T-cells can express integrin, allowing the adhesion to E-cadherin of the epithelium [48]. We speculate that high level of TGF-β induces EMT of DRs at the portal tract. These transitioning cells are capable of transdifferentiation into HPCs and movement towards injured sites, resembling chemotaxis. Since the number of immune cells and parenchymal cells decreases and results in the reduced TGF-β1 level at the lobule boundaries, the transitioning cells/HPCs may reverse EMT cascade and re-transdifferentiate into hepatic lineage to restore parenchyma [12]. The ultrastructural result that tonofilament bundles reoccurred in the cytoplasm of HPCs favors this opinion.

Non-resolving inflammation is established when the recruitment of inflammatory cells outstrips the mechanisms of resolution, including the apoptosis and the migration through lymphatics [49]. Non-resolving inflammation is responsible for abnormally elevated expression of TGF-β1 and the receptor for advanced glycation endproducts (RAGE), which plays an important role in the regulation of HPCs activation [50]. Consequently, most of the HPCs undergo complete EMT and differentiate into mature mesenchymal cells. The repair process is mainly fibrogenic because of the excess production and deposition of extracellular matrix components. The genetic or epigenetic changes may increase the susceptibility of HPCs to the detrimental microenvironment and result in self-renewing cells. Finally, HPCs or their progeny may transform to malignant cells because of the further accumulation of other alterations [50].

## Conclusion

Taken together, our study provided preliminary evidence that, at least in HBV-related hepatitis and cirrhosis, EMT is associated with the histogenesis and differentiation of HPCs. Ultrastructural results confirmed the direct interaction between HPCs and intermediate hepatocyte-like cells. The presence of tonofilament bundles in HPCs combined with double immunofluorescence staining results that they re-expressed epithelial markers CK7 and E-cadherin, rather than mesenchymal markers αSMA and vimentin, suggested that these transitioning HPCs may not complete the EMT cascade and launch the reverse process MET to gain epithelial features. However, this study mainly focused on human pathological tissues. Further experiments, especially in vitro and animal model studies, are required to explore the exact role of regulatory cytokines and microenvironment.

## Abbreviations

BM: Bone marrow
CK: Cytokeratin
DRs: Ductular reactions
EM: Electron microscopic
EMT/MET: Epithelial-mesenchymal transition/mesenchymal-epithelial transition
FSP1: Fibroblast-specific protein 1
H&E: Hematoxylin and eosin
HGF: Hepatocyte growth factor
HPCs: Hepatic progenitor cells
IHLCs: Intermediate hepatocyte-like cells
LCM: Laser capture microdissection
MMPs: Matrix metalloproteinases
OSM: Oncostatin M
RAGE: Advanced glycation endproducts
RT-qPCR: Quantitative reverse transcription PCR
αSMA: α-smooth muscle actin

## References

[1] Roskams T, De Vos R, Van Eyken P, Myazaki H, Van Damme B, Desmet V (1998). Hepatic OV-6 expression in human liver disease and rat experiments: evidence for hepatic progenitor cells in man. J Hepatol.

[2] Xiao JC, Ruck P, Kaiserling E (1999). Small epithelial cells in extrahepatic biliary atresia: electron microscopic and immunoelectron microscopic findings suggest a close relationship to liver progenitor cells. Histopathology.

[3] Gehling UM, Willems M, Dandri M, Petersen J, Berna M, Thill M (2005). Partial hepatectomy induces mobilization of a unique population of haematopoietic progenitor cells in human healthy liver donors. J Hepatol.

[4] Lemoli RM, Catani L, Talarico S, Loggi E, Gramenzi A, Baccarani U (2006). Mobilization of bone marrow-derived hematopoietic and endothelial stem cells after orthotopic liver transplantation and liver resection. Stem Cells.

[5] Yamada Y, Nishimoto E, Mitsuya H, Yonemura Y (2006). In vitro transdifferentiation of adult bone marrow Sca-1+ cKit- cells cocultured with fetal liver cells into hepatic-like cells without fusion. Exp Hematol.

[6] Roskams T, De Vos R, Desmet V (1996). Undifferentiated progenitor cells’ in focal nodular hyperplasia of the liver. Histopathology.

[7] Kaimori A, Potter J, Kaimori JY, Wang C, Mezey E, Koteish A (2007). Transforming growth factor-beta1 induces an epithelial-to-mesenchymal transition state in mouse hepatocytes in vitro. J Biol Chem.

[8] Nitta T, Kim JS, Mohuczy D, Behrns KE (2008). Murine cirrhosis induces hepatocyte epithelial mesenchymal transition and alterations in survival signaling pathways. Hepatology.

[9] Omenetti A, Porrello A, Jung Y, Yang L, Popov Y, Choi SS (2008). Hedgehog signaling regulates epithelial-mesenchymal transition during biliary fibrosis in rodents and humans. J Clin Invest.

[10] Kordes C, Sawitza I, Muller-Marbach A, Ale-Agha N, Keitel V, Klonowski-Stumpe H (2007). CD133+ hepatic stellate cells are progenitor cells. Biochem Biophys Res Commun.

[11] Yovchev MI, Grozdanov PN, Zhou H, Racherla H, Guha C, Dabeva MD (2008). Identification of adult hepatic progenitor cells capable of repopulating injured rat liver. Hepatology.

[12] Deng H, Wang HF, Gao YB, Jin XL, Xiao JC (2011). Hepatic progenitor cell represents a transitioning cell population between liver epithelium and stroma. Med Hypotheses.

[13] Wang H, Gao Y, Jin X, Xiao JC (2010). Expression of contactin associated protein-like 2 in a subset of hepatic progenitor cell compartment identified by gene expression profiling in hepatitis B virus-positive cirrhosis. Liver Int.

[14] Spee B, Carpino G, Schotanus BA, Katoonizadeh A, Vander Borght S, Gaudio E (2010). Characterisation of the liver progenitor cell niche in liver diseases: potential involvement of Wnt and Notch signalling. Gut.

[15] Van Den Heuvel MC, Slooff MJ, Visser L, Muller M, De Jong KP, Poppema S (2001). Expression of anti-OV6 antibody and anti-N-CAM antibody along the biliary line of normal and diseased human livers. Hepatology.

[16] Robertson H, Kirby JA, Yip WW, Jones DE, Burt AD (2007). Biliary epithelial-mesenchymal transition in posttransplantation recurrence of primary biliary cirrhosis. Hepatology.

[17] Okada H, Danoff TM, Kalluri R, Neilson EG (1997). Early role of Fsp1 in epithelial-mesenchymal transformation. Am J Physiol.

[18] Schmalhofer O, Brabletz S, Brabletz T (2009). E-cadherin, beta-catenin, and ZEB1 in malignant progression of cancer. Cancer Metastasis Rev.

[19] Thiery JP, Acloque H, Huang RY, Nieto MA (2009). Epithelial-mesenchymal transitions in development and disease. Cell.

[20] Kalluri R, Neilson EG (2003). Epithelial-mesenchymal transition and its implications for fibrosis. J Clin Invest.

[21] Casas E, Kim J, Bendesky A, Ohno-Machado L, Wolfe CJ, Yang J (2011). Snail2 is an essential mediator of Twist1-induced epithelial mesenchymal transition and metastasis. Cancer Res.

[22] Khan MA, Chen HC, Zhang D, Fu J (2013). Twist: a molecular target in cancer therapeutics. Tumour Biol.

[23] Peinado H, Ballestar E, Esteller M, Cano A (2004). Snail mediates E-cadherin repression by the recruitment of the Sin3A/histone deacetylase 1 (HDAC1)/HDAC2 complex. Mol Cell Biol.

[24] Bedossa P, Poynard T (1996). An algorithm for the grading of activity in chronic hepatitis C. The METAVIR Cooperative Study Group. Hepatology.

[25] Schmittgen TD, Livak KJ (2008). Analyzing real-time PCR data by the comparative C(T) method. Nat Protoc.

[26] Huber MA, Kraut N, Beug H (2005). Molecular requirements for epithelial-mesenchymal transition during tumor progression. Curr Opin Cell Biol.

[27] Egeblad M, Werb Z (2002). New functions for the matrix metalloproteinases in cancer progression. Nat Rev Cancer.

[28] Radisky DC, Levy DD, Littlepage LE, Liu H, Nelson CM, Fata JE (2005). Rac1b and reactive oxygen species mediate MMP-3-induced EMT and genomic instability. Nature.

[29] Schneider MR, Hiltwein F, Grill J, Blum H, Krebs S, Klanner A (2014). Evidence for a role of E-cadherin in suppressing liver carcinogenesis in mice and men. Carcinogenesis.

[30] Nakagawa H, Hikiba Y, Hirata Y, Font-Burgada J, Sakamoto K, Hayakawa Y (2014). Loss of liver E-cadherin induces sclerosing cholangitis and promotes carcinogenesis. Proc Natl Acad Sci U S A.

[31] Means AL, Xu Y, Zhao A, Ray KC, Gu G (2008). A CK19(CreERT) knockin mouse line allows for conditional DNA recombination in epithelial cells in multiple endodermal organs. Genesis.

[32] Schneider MR, Kolligs FT (2015). E-cadherin's role in development, tissue homeostasis and disease: Insights from mouse models: Tissue-specific inactivation of the adhesion protein E-cadherin in mice reveals its functions in health and disease. Bioessays.

[33] Yang J, Mani SA, Donaher JL, Ramaswamy S, Itzykson RA, Come C (2004). Twist, a master regulator of morphogenesis, plays an essential role in tumor metastasis. Cell.

[34] Martin TA, Goyal A, Watkins G, Jiang WG (2005). Expression of the transcription factors snail, slug, and twist and their clinical significance in human breast cancer. Ann Surg Oncol.

[35] Huang KT, Dobrovic A, Yan M, Karim RZ, Lee CS, Lakhani SR (2010). DNA methylation profiling of phyllodes and fibroadenoma tumours of the breast. Breast Cancer Res Treat.

[36] Vesuna F, van Diest P, Chen JH, Raman V. Twist is a transcriptional repressor of E-cadherin gene expression in breast cancer. Biochem Biophys Res Commun. 2008;367(2):235–41. doi:S0006-291X(07)02575-2 10.1016/j.bbrc.2007.11.151. 10.1016/j.bbrc.2007.11.151 PMC2696127 18062917

[37] Vincent T, Neve EP, Johnson JR, Kukalev A, Rojo F, Albanell J (2009). A SNAIL1-SMAD3/4 transcriptional repressor complex promotes TGF-beta mediated epithelial-mesenchymal transition. Nat Cell Biol.

[38] Venkov CD, Link AJ, Jennings JL, Plieth D, Inoue T, Nagai K (2007). A proximal activator of transcription in epithelial-mesenchymal transition. J Clin Invest.

[39] Taura K, Miura K, Iwaisako K, Osterreicher CH, Kodama Y, Penz-Osterreicher M (2010). Hepatocytes do not undergo epithelial-mesenchymal transition in liver fibrosis in mice. Hepatology.

[40] Zeisberg M, Yang C, Martino M, Duncan MB, Rieder F, Tanjore H (2007). Fibroblasts derive from hepatocytes in liver fibrosis via epithelial to mesenchymal transition. J Biol Chem.

[41] Valdes F, Alvarez AM, Locascio A, Vega S, Herrera B, Fernandez M (2002). The epithelial mesenchymal transition confers resistance to the apoptotic effects of transforming growth factor Beta in fetal rat hepatocytes. Mol Cancer Res.

[42] Yimlamai D, Christodoulou C, Galli GG, Yanger K, Pepe-Mooney B, Gurung B (2014). Hippo pathway activity influences liver cell fate. Cell.

[43] Anzai K, Chikada H, Tsuruya K, Ida K, Kagawa T, Inagaki Y (2016). Foetal hepatic progenitor cells assume a cholangiocytic cell phenotype during two-dimensional pre-culture. Sci Rep.

[44] Yang P, Li QJ, Feng Y, Zhang Y, Markowitz GJ, Ning S (2012). TGF-beta-miR-34a-CCL22 signaling-induced Treg cell recruitment promotes venous metastases of HBV-positive hepatocellular carcinoma. Cancer Cell.

[45] Rygiel KA, Robertson H, Marshall HL, Pekalski M, Zhao L, Booth TA (2008). Epithelial-mesenchymal transition contributes to portal tract fibrogenesis during human chronic liver disease. Lab Invest.

[46] Askenasy N, Kaminitz A, Yarkoni S (2008). Mechanisms of T regulatory cell function. Autoimmun Rev.

[47] Rehermann B (2013). Pathogenesis of chronic viral hepatitis: differential roles of T cells and NK cells. Nat Med.

[48] Cepek KL, Shaw SK, Parker CM, Russell GJ, Morrow JS, Rimm DL (1994). Adhesion between epithelial cells and T lymphocytes mediated by E-cadherin and the alpha E beta 7 integrin. Nature.

[49] Nathan C, Ding A (2010). Nonresolving inflammation. Cell.

[50] Pusterla T, Nemeth J, Stein I, Wiechert L, Knigin D, Marhenke S (2013). Receptor for advanced glycation endproducts (RAGE) is a key regulator of oval cell activation and inflammation-associated liver carcinogenesis in mice. Hepatology.

